# Home Monitoring of High-Risk Individuals Receiving Opioids Post Orthopedic Surgery

**DOI:** 10.1089/respcare.11783

**Published:** 2025-08-04

**Authors:** Robert L. Mazzola, Kim J. Bennion, Megan J. Hepworth, Greg G. Petersen, Gaylinn Breeze, Kelly C. Jensen, Megan Jensen, Vanessa Henriksen, Lisa Bagley, Chance Keddington, Gregory L. Snow, Tom Belnap, Carrie M. Winberg, Shawna B. Papenfuss, Tom V. Cloward

**Affiliations:** ^1^ Drs. Mazzola and Cloward are affiliated with Department of Sleep Medicine, Intermountain Health Care Inc, Murray, Utah, USA.; ^2^ Mss. Bennion, Hepworth, Mr. Jensen, Ms. Jensen, and Ms. Winberg are affiliated with Department of Respiratory Care, Intermountain Health Care Inc, Murray, Utah, USA.; ^3^ Dr. Petersen is affiliated with Department of Anesthesia, Intermountain Health Care Inc, Murray, Utah, USA.; ^4^ Dr. Breeze is affiliated with Department of Medicine, Intermountain Health Care Inc, Murray, Utah, USA.; ^5^ Ms. Henriksen is affiliated with Office of Patient Experience, Intermountain Health Care Inc, Murray, Utah, USA.; ^6^ Ms. Bagley is affiliated with Department of Administration, Intermountain Health Care Inc, Murray, Utah, USA.; ^7^ Dr. Keddington is affiliated with Department of Pharmacy, Intermountain Health Care Inc, Murray, Utah, USA.; ^8^ Dr. Gregory is affiliated with Department of Statistics, Brigham Young University, Provo, Utah, USA.; ^9^ Mr. Belnap is affiliated with Department of Enterprise Analytics, Intermountain Health Care Inc, Murray, Utah, USA.; ^10^ Dr. Papenfuss is affiliated with Department of Education, Intermountain Health Care Inc, Murray, Utah, USA.

**Keywords:** ambulatory surgery, capnography, home monitoring, obstructive sleep apnea, opioids, pulse oximetry, quality improvement, respiratory depression, respiratory insufficiency

## Abstract

**Background:**

Postoperative patients receiving opioids are at risk for morbidity and mortality caused by opioid-induced respiratory depression (OIRD). Guidelines advocate electronic monitoring for OIRD during postoperative hospitalization, but the utility of home monitoring following ambulatory surgery has not been assessed. We describe the utilization of capnography and pulse oximetry in an ambulatory orthopedic cohort to determine subject/home caregiver acceptance and utility of continuous monitors at home. The specific aims of this initiative were to (1) determine the subject/home caregiver acceptance of home monitoring to detect OIRD in patients after hospital discharge following orthopedic surgery, (2) determine the rate of OIRD and associated morbidity after hospital discharge following orthopedic surgery, and (3) determine patient understanding of the risk of OIRD.

**Methods:**

This prospective, subject/home caregiver acceptance quality improvement initiative was conducted from September 28, 2019, to October 31, 2020. Ambulatory subjects undergoing orthopedic surgical procedures had cardiorespiratory data monitored with a commercially available device at home for 4 days while napping/sleeping. Recorded data were analyzed for reliability comparing end-tidal carbon dioxide pressure (P_ETCO_2__), S_pO_2__, breathing frequency (f), and heart rate (beats/min).

**Results:**

Three hundred fifty-nine subjects were enrolled and had complete data. Two hundred fifty-two (70%) were discharged with supplemental oxygen. When comparing audible alarms with/without delays, there were 4,770/22,409 low P_ETCO_2__, 1,601/6,246 high P_ETCO_2__, 460/4,211 low frequency, 1,572/6,547 low heart rate, and 462/5,520 low S_pO_2__ alarms. Twenty-six (7.2%) subjects visited the emergency department in response to low S_pO_2__ audible alarms. Of these, 14 (3.9%) were diagnosed with a clinically relevant opioid-induced event, 95% CI 2.1%–6.5%, and 1 subject was administered naloxone by home caregivers.

**Conclusions:**

This study demonstrates home monitoring of oxygenation (S_pO_2__), but not respiration (P_ETCO_2__), following ambulatory orthopedic procedures is feasible. Subjects at risk for clinically relevant opioid events may experience higher rates of postoperative complications, hospital readmissions, clinically relevant events, or death.

## Introduction

Opioids are potent analgesics that are commonly used for the treatment and adequate relief of pain, including postoperative moderate-to-severe pain. However, opioids have several adverse effects, of which respiratory depression (defined as < 8 to 10 breaths/min) is a potentially dangerous and life-threatening adverse event. Postoperative opioid-induced respiratory depression (OIRD) is a serious complication that has spurred medical societies and professional councils to issue guidance for presurgical screening and postoperative monitoring of hospital in-patients.^1–6^ Factors that contribute to high risk for OIRD have been described in literature, including advanced age, hypertension, increased body mass index, obstructive sleep apnea, COPD, congestive heart failure, renal failure, hemodialysis/peritoneal dialysis, and opioids prescribed for acute or chronic pain where total morphine milliequivalents per day may exceed safe dosing standards.

The total number of ambulatory surgeries in the United States increased from an estimated 380,000 in 1983 and 31.5 million in 1996 to an estimated 53.3 million in 2006.^7,8^ In particular, orthopedic procedures are increasingly being performed on an ambulatory basis. Many of these patients have opioid analgesia requirements. The utilization of nerve blocks may also be required in knee, hip, and shoulder replacements, which are often performed as same-day surgeries.^8^

Lee et al performed an anesthesia closed claims malpractice analysis of postoperative OIRD to determine if injuries could guide preventive strategies.^9^ OIRD was judged as possible, probable, or definitive. Ninety-two claims were preventable, with 88% occurring within the first 24 h of surgery. Over half the subjects were not on respiratory monitors, and somnolence occurred in 60% of subjects prior to respiratory depression that went unrecognized. Worrisome is that the first 24 postoperative hours have been reported as the highest risk for life-threatening OIRD episodes in adults.^10–14^ Chung et al demonstrated that monitoring patients postoperatively for respiratory depression may require more than 1–2 nights postoperatively. Outcomes showed the apnea-hypopnea index in subjects was highest on the third night post-surgery and remained above the preoperative baseline as far as the seventh night.^15^

Societies and professional councils agree on the need for assessment of sedation level, continuous monitoring of oxygenation/ventilation, and early response and intervention in the first 24 h postoperatively to prevent OIRD.^1–3,5,16^ Postoperative monitoring with pulse oximetry alone and pulse oximetry with capnography have been described on general care wards.^5,17–19^ Both pulse oximetry and capnography are well established and recommended monitoring modalities that have been well described in literature. However, use as continuous monitoring technology at home following ambulatory surgery has not been described. Guidelines specific to ambulatory procedures are lacking regarding monitoring of oxygenation and ventilation following discharge from the surgical center.^2^

Sun et al and Taenzer studied pulse oximetry independently.^17,18^ There is continued debate in the effectiveness of pulse oximetry alone.^17,18^ Capnography with pulse oximetry may be more sensitive than pulse oximetry alone.^5,19^ These parameters have been studied in operating rooms and hospital units; however, we could find no studies utilizing capnography and/or pulse oximetry for postoperative subjects in the home setting.

The Society for Ambulatory Anesthesia published a consensus statement in 2015 of guidelines for ambulatory surgery subjects with obstructive sleep apnea.^2^ A systematic review of subjects with obstructive sleep apnea, subjects with low-risk obstructive sleep apnea, and subjects with nonobstructive sleep apnea was performed. Several recommendations were contradictory to the 2006 American Society for Anesthesia obstructive sleep apnea guidelines. Non-adherence to positive airway pressure therapy is considered a risk for poor outcomes during preoperative evaluation.^2,15^ Prescreening for potential obstructive sleep apnea with the STOPBANG questionnaire has become an accepted practice.^20^ The STOPBANG questionnaire is an 8-item screening tool for the risk of obstructive sleep apnea consisting of questions on snoring, tiredness, observed apnea, high blood pressure, body mass index, age, neck size, and gender. Studies regarding the efficacy of new-generation pulse oximeters in terms of accuracy during motion artifact, low perfusion, ability to read through varying skin pigmentations, and signal integrity are numerous.^21–26^ These elements were taken into consideration when selecting home monitoring equipment for this study.

Patients prescribed higher opioid dosages are at higher risk for mortality and morbidity.^3^ A 2004–2009 study performed by the Veteran’s Health Administration reported 1,877,841 participants with opioid prescriptions for chronic pain with an average of 98 morphine milliequivalents per day had increased complications.^27^ In 2016, the Centers for Disease Control and Prevention published 12 key guidelines for opioid prescribing in chronic pain.^28^ One key recommendation was “clinicians should use caution when prescribing opioids at any dosage and should carefully reassess evidence of individual risks/benefits when considering increasing dosage to ≥ 50 morphine milligram equivalents per day and should avoid increasing dosage ≥ 90 morphine milligram equivalents per day or carefully justify a decision to titrate dosage to ≥ 90 morphine milligram equivalents per day” (recommendation category: A, evidence type: 3).

The specific aims of this study, therefore, are to (1) determine the feasibility of home monitoring to detect OIRD in subjects after hospital discharge following orthopedic surgery, (2) determine the rate of OIRD and associated morbidity after hospital discharge following orthopedic surgery, and (3) determine patient understanding of the risk of OIRD. Our hypothesis was that monitoring technology would be feasible in the home environment and would detect clinically relevant OIRD events.

**QUICK LOOK**

**Current knowledge**

Postoperative opioid-induced respiratory depression (OIRD) is a serious complication especially in patients identified as high-risk for death from opioids. Factors that contribute to high risk for OIRD include advanced age, hypertension, increased body mass index, obstructive sleep apnea, COPD, congestive heart failure, renal failure, hemodialysis/peritoneal dialysis, and opioid overdose. Medical societies have issued guidance for presurgical screening and postoperative monitoring of high-risk patients. Current literature is limited regarding the identification of opioid high-risk patients who would benefit from home monitoring especially in same-day discharged, postoperative, orthopedic patients.

**What this paper contributes to our knowledge**

In this relatively small cohort of postoperative, orthopedic subjects, home monitoring detected clinically relevant OIRD events resulting in subjects seeking care in emergency departments. Monitoring alarm thresholds and delays improved subjects’ perception of actionable alarms. The accuracy of end-tidal carbon dioxide pressure was impaired by condensation in the sampling line in the uncontrolled home environment. Continuous S_pO_2__ monitoring was feasible for home monitoring and was a indicator of early respiratory depression.

## Methods

This prospective, quality improvement initiative was conducted in accordance with the ICH Good Clinical Practice E6(R2) guidelines, the Code of Federal Regulations, and the Declaration of Helsinki. A request for a waiver of the written informed consent was granted by the Intermountain Health Institutional Review Board (IRB #1051133, ref #018611, approved on July 31, 2019). Approval of the written informed consent waiver was based on the intent of this study for quality improvement of health care operations and compliance with The Privacy Rule, 45 Code of Federal Regulations 164.506. Only verbal consent was required and was defined as the subject’s agreement to enroll. The Intermountain Medical Research Foundation, the Orthopedic Specialty Hospital, and Pharmacy Clinical Services funded the study but had no input into any part of the study design, any aspect of outcomes, or manuscript review.

This prospective, quality improvement, open, cohort initiative included adult ambulatory and in-patient orthopedic surgical subjects. Investigators used a quantitative methodology with a descriptive nonrandomized, prospective, single cohort design. The primary study outcome was the determination of the subject/home caregiver acceptance of home monitoring of respiration and oxygenation for the identification of OIRD and other clinically relevant events. Capnography with end-tidal carbon dioxide pressure (P_ETCO_2__) and pulse oximetry (S_pO_2__) with and without audible alarm delays were compared.

Secondary outcomes of hospital-specific quality practices were evaluated retrospectively in terms of (1) presurgical capture of adherence to prescribed positive airway pressure therapy in subjects diagnosed with obstructive sleep apnea prior to surgery, (2) oxygen desaturation index per hour postoperatively in subjects not previously diagnosed with sleep apnea, (3) physician-prescribed morphine milligram equivalents per day postoperatively for pain, and (4) frequency of subjects discharged with home oxygen postoperatively who did not require it before surgery. Outcomes were captured with pre-/post-study surveys (eg, overall subject engagement and feedback), recorded monitor outcomes (eg, oxygen desaturation index per hour, S_pO_2__, P_ETCO_2__, breathing frequency, and heart rate in beats/min trending), and chart reviews (eg, surgery type, nerve blocks, morphine milligram equivalents, emergency department visits, and diagnoses).

This initiative was conducted in a 40-bed ambulatory and in-patient surgical hospital specialized in orthopedic surgery (The Orthopedic Specialty Hospital) and in subjects’ homes. Adult patients seen at the Intermountain the Orthopedic Specialty Hospital and clinics were considered for inclusion if they met the high risk for OIRD criteria as defined in the protocol. Subjects included were ≥18 years of age who were undergoing a same-day orthopedic surgical procedure as well as those who were admitted to the hospital. Preoperative screening was conducted in the surgeon’s office and in a dedicated perioperative screening clinic prior to surgery. Physiologic state, comorbid conditions to include previous and suspected obstructive sleep apnea diagnosis, and required preoperative evaluations, including the STOPBANG score in patients not previously diagnosed with obstructive sleep apnea, were assessed for appropriateness of surgery at the facility. Inclusion criteria, which identified the criteria for the definition “high-risk subjects,” were defined per protocol in Box 1 of the study protocol (available in Supplementary Data S1). “High-risk subjects” were defined as (1) STOPBANG score ≥5 (no previous sleep study, no home use of CPAP, noninvasive ventilation, or auto-titrate positive airway pressure); (2) previous diagnosis of obstructive sleep apnea and non-adherent with positive airway pressure therapy or positive airway pressure therapy not yet ordered; (3) subject required positive airway pressure therapy in the post-anesthesia care unit or on a patient care floor who was not utilizing it at home prior to procedure; (4) the presence of recurrent respiratory event in the nonstimulated subject (defined by S_pO_2__ < 90%, bradypnea < 8 breaths/min, and/or apnea >10 s); (5) pain/sedation mismatch (high pain and sedation scores concurrently); (6) subjects requiring supplemental oxygen who did not require it pre-procedure/pre-hospital admission; and/or (6) extended post-anesthesia care unit recovery time ≥ 90 min. Only patients meeting the inclusion criteria were approached for enrollment. Patients were excluded from participation if they were pregnant or lactating females, or they were unwilling to use the home monitoring device following hospital discharge.

Subjects who met the eligibility criteria were presented with the initiative’s information, and they were provided with as much time as they need to consider participation, ask questions, and obtain satisfactory answers to their questions. Subjects who verbally agreed to participate were included. Following their verbal agreement to participate, subjects viewed a 4-min video (“Parker’s Video”) about a 22-year-old patient who died from OIRD following a routine, uncomplicated tonsillectomy after taking opioids in half the dose prescribed. This video was loaded on a portable tablet for ease of subject’s viewing and was also available online (https://www.youtube.com/watch?v=R-4JwdUC4h0). The goal was to raise the subjects’ awareness of OIRD that can happen even when taking opioids as prescribed.

Participating subjects received a commercially available home respiratory monitor capable of recording continuous P_ETCO_2__, breathing frequency, heart rate, and S_pO_2__. Selection of the device was based on portability, S_pO_2__ accuracy in lieu of skin pigmentation, accuracy despite artifact and/or poor perfusion, decreased false alarming, and the ability to additionally monitor P_ETCO_2__, heart rate, and breathing frequency.^6,21–24^ Participants also received training in the use of the device and response to device alarms to be monitored during napping and sleeping for a total of 4 days postoperatively. Subjects were discharged with nasal spray naloxone. Pharmacy staff provided instructions regarding nasal spray naloxone indications and dispensing. A patient diary and instructions for completing it were provided. Postoperative subjects who agreed to participate received the monitoring device upon discharge.

Upon discharge, subjects and home caregivers were educated on monitor setup, use, alarms, interventions, and the overall risk of OIRD. They were instructed to wear the monitor while napping and sleeping, with recorded data being obtained for 96 h (4 d) after hospital discharge.^3,5,18,25^ Monitors were returned to Intermountain where data download, scrubbing, and analysis were done as close to the same day as the equipment return as possible. Enrolled subjects and home caregivers were provided education verbally with teach-back demonstrations. A study folder of printed instructions was provided, including a “score card” for troubleshooting guidance, instructions for responding to alarms, indications for calling 911, and nasal naloxone administration. Subjects were discharged with 2 doses of nasal naloxone. Subjects were provided with a 24-h telephone number that was supported by registered respiratory therapists. Registered respiratory therapists were available for questions and concerns and could escalate issues and problems to advanced practice providers. Education regarding the study discharge process was given to hospital staff with case simulations being conducted prior to the start of the home monitoring initiative.

**Fig. 1.**
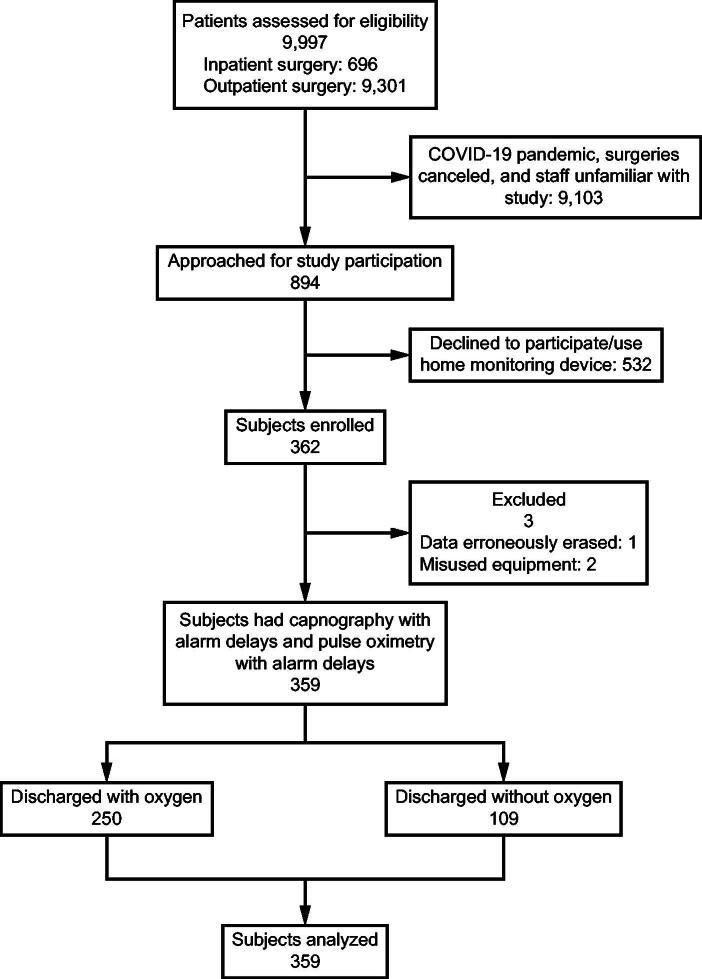
Flow chart.

All subjects who were discharged on home oxygen met the Centers for Medicare and Medicaid criteria for the medical necessity for home oxygen per room air S_pO_2__ at <88%. Subjects requiring oxygen postoperatively who did not require it prior to the procedure were considered high risk for opioid use. This was included in the inclusion criteria for this initiative as identified in Figure 1. For the primary aim of this initiative, the subject/home caregiver acceptance of home monitoring for the detection of OIRD in subjects after discharge following orthopedic surgery was assessed by a review of the subject responses to 2 questionnaires that the subjects completed. Subjects completed the first questionnaire prior to discharge from the hospital and the second questionnaire upon return of the home monitoring equipment. The first questionnaire consisted of 11 questions (answerable by “yes” or “no”) about understanding the rationale for home monitoring and instructions on use of the monitoring equipment; previous use of opiate medications and home oxygen; history of surgical procedures, anesthesia use, and breathing difficulties; awareness of OIRD; and the adequacy of the pre-discharge education and instructions that they received. The second questionnaire consisted of 14 questions (answerable by “yes” or “no” and open-text responses) about the subject’s use of the home monitoring equipment, any associated problems, and their perception of safety while on home monitoring; response to alarms and any actions taken; number of calls to the hotline number and actions taken; any visits to the emergency department; use of naloxone; and their willingness to use a home monitoring equipment in future. Verbal/telephone data capture occurred if subjects failed to complete and return the post-study survey with the monitoring equipment. Questions were asked of subjects verbatim as written on the survey tool. The rate of OIRD was determined from a review of each subject’s medical records. The subjects’ associated morbidity events after hospital discharge following orthopedic surgery were determined by a review of the participating subjects’ medical records.

Data were obtained from the subjects’ electronic medical records, laboratory and procedural test reports, responses to 2 written questionnaires, and/or directly from the subjects via telephone calls. Data were downloaded and recorded in the database by a member of the initiative oversight staff (ie, respiratory care student interns with oversight by the enterprise Administrative Director of Respiratory Care) using the home monitoring device. Subject demographics, comorbid conditions, surgical course, anesthesia type, the use of peripheral nerve blocks (ie, interscalene, femoral, and sciatic), and opioids for pain prescribed and filled at an Intermountain Health pharmacy (defined as morphine milligram equivalents per day) were included. The monitoring device software data were utilized for extraction of S_pO_2__, P_ETCO_2__, frequency, and heart rate trending, alarms, and calculation of the oxygen desaturation index per hour. Responses to the questionnaires were analyzed, and the data were entered into the study database by the respiratory therapy student interns with oversight by the enterprise Administrative Director of Respiratory Care. Study data were stored and shared with the initiative team leads via a commercially available web-based electronic data capture tool that supports clinical and research studies. The postoperative course was evaluated for emergency department visits, naloxone administration, death, or other clinically relevant OIRD and non-OIRD-related events. These were determined by visits to the emergency department at 4 days, 30 days, and 60 days post-discharge if opioids were still being prescribed. Events caused by opioids were defined by those documented in the medical record as such (eg, opioid-induced organ failure owing to hypoxemia). Surveys pre- and post-study participation were conducted by registered respiratory therapists to capture positive airway pressure adherence as compared with physician clinic/nursing preoperative assessment, subject knowledge of OIRD pre-surgery, subject/home caregiver response to various aspects of the monitoring supplies, ease of monitor use, response to alarms, and overall engagement with the monitors and study.

Outcomes were ascertained through electronic interrogation of the home monitor devices (P_ETCO_2__, frequency, S_pO_2__, OIRD episodes, alarm instances, time spent with device operating), pre- and post-intervention survey of participating patients (satisfaction with device use, understanding of opioid-related risks, response to alarms, use of naloxone); and electronic review of patient medical records (hospital or emergency department visits during the study period). High risk for obstructive sleep apnea was defined as the presence of 5 or more criteria from the STOPBANG screening tool as decided by the enterprise Anesthesiology Development Team. Subject groups analyzed were subjects monitored with capnography with P_ETCO_2__ versus pulse oximetry with S_pO_2__ with and without alarm delays and patients discharged with versus without oxygen supplementation. To assess physician opioid prescribing practice, a retrospective chart review was completed to determine surgery type, nerve blocks, and prescribed opioid morphine milligram equivalents per day for pain.

Alarm parameters included high/low limits with audible alarm delays to avoid alarm fatigue. Frequency and heart rate were captured by the home monitoring device. The breathing frequency high alarm was set to off, and the low was set for ≤6 breaths/min with a 30-s delay. The heart rate high alarm was off with the low alarm set at ≤50 beats/min with a 20-s delay. S_pO_2__ high alarm was set to off with the low alarm set at ≤85% with a 30-s delay. P_ETCO_2__ high alarm was set at 50 mm Hg, and the low was set at 20 mm Hg with a delay of 15 s for both.

Using R statistical software (https://www.r-project.org/), variables for each group were analyzed using mean and standard deviation, median and interquartile range, or counts and percentages as appropriate. Tests to compare the variables of interest included the Welch modified *t* test (does not assume equal variances) or the Wilcoxon rank-sum test for continuous variable depending on the amount of skewness in the data and chi-square or Fisher exact test for categorical data (depending on expected cell counts). This initiative is mostly exploratory, and each test was of specific interest rather than attempting to declare success if any of several tests was found significant. We did not perform any adjustments for multiple testing. The goal of 500 patients limited the margin of error to <5%. The margin of error was more relevant than specific power.

## Results

Subject screening, enrollment, and analysis groups are shown in the CONSORT diagram in Supplementary Data S1. Between September 28, 2019, and October 31, 2020, 9,997 patients were identified as meeting the study inclusion criteria. The COVID-19 pandemic interrupted the study when elective surgeries were cancelled and staff was shared from other hospitals who were not familiar with the study. This resulted in only 894 patients being approached for enrollment, of which 532 refused participation and/or were unwilling to wear the home monitoring device. Table 1 shows the demographic information of all subjects where data are reported comparing subjects discharged home with/without oxygen. Utilizing both chi-square and Fisher exact test, no significance was noted when comparing the gender of subjects enrolled (*P* = .26). Subjects discharged with oxygen were slightly older than those discharged without oxygen (Wilcoxon *P* value = .002). There was no significant difference in body mass index between subjects discharged with and without oxygen (Wilcoxon *P* value = .11).

**Table 1.** Subject demographics

Total (*N =* 359)	Subjects with home oxygen (*n =* 250 [70%])	Subjects without home oxygen (*n =* 109 [30%])
Gender		
Male	126 (50)	61 (56)^a^
Female	124 (50)	45 (41)^a^
Age in years		
Median	63.3 (IQR 15.8)	58.7 (IQR 18.8)
<18	1 (<1)	0 (0)
18–30	2 (<1)	5 (5)
31–50	39 (16)	27 (25)
51–65	107 (43)	45 (41)
>65	101 (40)	29 (26)
Not reported	0 (0)	3 (3%)
Race		
White	245 (98)	96 (88)
Asian	0 (0)	2 (2)
Hawaiian/Pacific Islander	1 (<1)	1 (<1)
Unavailable	3 (<1)	10 (10)
Other/subject declined to declare	5 (2.0)	13 (11.9)
Emergency department or urgent care visit	15 (6.0)	10 (9.1)
History of obstructive sleep apnea	76 (30.4)	52 (47.7)
Positive airway pressure adherent	2 (<1.0)	0 (0)
Oral device	1 (<1.0)	0 (0)
Unknown	7 (2.8)	1 (<1.0)
Diabetes diagnosis	41 (16.4)	16 (14.7)
Sleep study post-study participation	15 (6.0)	3 (2.8)
Body mass index (kg/m^2^)		
18.5–29	80 (32.0)	38 (34.9)
30–55	170 (68.0)	64 (58.1)
Surgery type		
Arthroplasty hip	17 (6.8)	5 (4.6)
Arthroplasty knee	71 (28.4)	11 (10.1)
Arthroplasty shoulder	29 (11.6)	4 (3.7)
Spine	33 (13.2)	18 (16.5)
Arthroscopy	52 (20.8)	32 (29.4)
Other	48 (19.2)	39 (35.8)
Anesthesia type		
General	166 (66.4)	81 (74.3)
Spinal	84 (33.6)	20 (18.4)
Monitored anesthesia care/sedation	3 (<1.0)	5 (4.6)
Nerve blocks (femoral, interscalene, sciatic)	89 (35.6)	21 (19.3)
None	161 (64.4)	85 (78.0)
STOP-BANG score ranges		
1–2 (low risk)	51 (20.4)	6 (5.5)
3–4 (medium risk)	68 (27.2)	12 (11.0)
5–8 (high risk)	60 (24.0)	26 (23.9)
Not scored: previous OSA diagnosis	71 (28.4)	65 (59.6)
Morphine milliequivalents per day for home discharge (prescriptions filled)		
≤50	40 (16.0)	22 (20.2)
51–89	37 (14.8)	21 (19.3)
≥90–119	78 (31.2)	34 (31.2)
≥120	62 (24.8)	10 (9.2)
Not applicable	0 (0)	3 (2.8)
Discharged with 2 opioid pain prescriptions by morphine milliequivalents per day		
135 and 60	1 (<1)	0 (0)
30 and 30	1 (<1)	0 (0)
135 and 144	1 (<1)	0 (0)

^a^ Subjects discharged home without oxygen reported 3 (3%) with gender and age not reported.

Data shown as *n* (%) unless otherwise noted. IQR, interquartile range; OSA, obstructive sleep apnea.

One hundred twenty-eight (36%) subjects in the study population were previously diagnosed with sleep apnea and prescribed positive airway pressure therapy or oral appliance for treatment. Only 3 (2%) subjects were adherent with the ordered obstructive sleep apnea positive airway pressure therapy (*P* < .001).

Prior internal audits reported 19–24% of The Orthopedic Specialty Hospital patients had been discharged home on supplemental oxygen postoperatively who had not required it prior to surgery. Within the study population, 250 (70%) were discharged with home oxygen, which was one of several possible study inclusion criteria. The authors recognize the bias toward oxygen use; therefore, subjects discharged with/without oxygen were compared separately. When comparing sex aggregately, females with/without supplemental oxygen with males with/without supplemental oxygen, there was statistical significance for more women requiring supplemental oxygen at discharge (*P* = .02).

The oxygen desaturation index is the hourly average of desaturation events defined in this study as a 3% decrease in S_pO_2__ for ≥10 s and is reported as the number of events per hour. The oxygen desaturation index has been used as a predictor for sleep-disordered breathing, which can be elevated with concurrent opioid use. Twenty-five (7%) of the total study population experienced ≥50 oxygen desaturation index per hour. Ten or greater oxygen desaturation index per hour have been correlated with a greater chance of sleep-disordered breathing because of airway obstruction. When analyzing aggregate oxygen desaturation index per hour using the Welch 2-sample *t* test and comparing those with/without home oxygen, there was statistical significance in subjects with oxygen (*P* = .02). This may be underestimated in the group with supplemental oxygen, which can attenuate hypoxemia and desaturation events. There was no statistical significance in the oxygen desaturation index when comparing sex alone (*P* = .15). There was no statistical significance in the oxygen desaturation index being elevated while on supplemental oxygen and adjusting for sex (*P* = .37). Oxygen desaturation index greater than 10 events per hour did not correlate with subjects visiting the emergency department or requesting further evaluation for sleep-disordered breathing. Subjects undergoing general anesthesia had greater oxygen desaturation index scores (*P* = .053).

When comparing subjects discharged with and without oxygen who received nerve blocks, 89 (36%) discharged received nerve blocks compared with 21 (20%) who did not receive nerve blocks (Fisher exact test *P* = .004). Subjects receiving interscalene nerve blocks with >90 morphine milligram equivalents required increased need for supplemental oxygen, which was found to be statistically significant (*P* = .001).

Included in the study were 137 (39%) surgical arthroplasties of the hip, knee, and shoulder. Fifty-one (14%) spinal procedures, 84 (23%) arthroscopy procedures, and 87 (24%) were included as other procedures. Anesthetic variables included 247 (69%) of subjects receiving general anesthesia, 104 (29%) receiving spinals, and 8 (2%) receiving monitored anesthesia care.

Of the 306 subjects discharged with pain medications ordered and filled at an Intermountain Health pharmacy, 241 (79%) received >50 morphine milligram equivalents per day. In subjects with nerve blocks, the need for supplemental oxygen was higher in all ranges of morphine milligram equivalents compared with subjects not administered nerve blocks. This was most notable in subjects with 90–120 morphine milligram equivalents and >120 morphine milligram equivalents per day. The Pearson’s chi-square test reported a *P*-value < .001. Subjects receiving interscalene nerve blocks with >90 morphine milligram equivalents required an increase in supplemental oxygen, which was statistically significant (*P* < .001).

Of the total 359 subjects enrolled in the study, 358 (99.7%) completed the pre-study survey. Of these, 346 (95%) reported feeling more informed about the risks of taking opioid after watching Parker’s video. Seventy (19%) subjects wore the monitor for the 4 days as instructed, with 16 (5%) choosing to wear the monitor for up to 11 days. The mean value of 2.2 days (SD 1.8) of monitor use supported subjects beyond the first 24 h postoperatively. Subjects reported responding predominantly to the low S_pO_2__ alarms. All subjects who visited the emergency department reported the low S_pO_2__ alarm was the triggering factor.

High and low alarm settings, alarm delays, and the number of alarms with/without delays were compared and are reported in Table 2. There were 6,371 low alarms for P_ETCO_2__, to which subjects responded. When comparing alarms with/without delays, there would have been 28,655 low alarms for P_ETCO_2__ had the delay not been set. These were predominately triggered by condensation in the cannula line. Subjects reported the low P_ETCO_2__ alarm would sound and awaken them only to find the P_ETCO_2__ was reading “0” whereas S_pO_2__ was within normal limits. The authors noted condensation in the capnography lines after visiting subjects in their homes who complained of P_ETCO_2__ over alarming. Of the 293 (82%) subjects completing the post-study surveys, 217 (61%) would participate again in home monitoring postoperatively and 135 (38%) felt safer with monitor use. One hundred forty-five (40%) home caregivers felt safer when subjects were monitored. One hundred seventy subjects (47%) felt there were too many alarms with end-tidal capnography at the top of the list. Subjects/caregivers reported S_pO_2__ alarm to be the most reliable.

**Table 2.** Device monitor alarms with/without delay settings

Alarm type	Alarm setting	Alarm delay	Total actual alarms subjects received with set delays, no.	Total alarms subjects would have received without set delays, no.
S_pO_2__ high	Off	Off	0	0
S_pO_2__ low	≤85%	30 s	462	5,520
Breathing frequency high^a^	Off	Off	0	0
Frequency low^a^	≤6 breaths/min	30 s	460	4,211
Heart rate high	Off	Off	0	0
Heart rate low	≤50 beats/min	20 s	1,572	64,57
P_ETCO_2__ high^a^	50 mm Hg	15 s	1,601	6,246
P_ETCO_2__ low^a^	20 mm Hg	15 s	4,770	22,409

^a^ Low P_ETCO_2__ and frequency alarms were felt to be unreliable as the sampling line condensation triggered inaccurate readings and audible alarms.

BPM, beats/min; mm Hg, millimeters of mercury; P_ETCO_2__, end-tidal carbon dioxide pressure.

Excluding low P_ETCO_2__, the most common alarm was low S_pO_2__. Low S_pO_2__ levels prompted 26 (7.2%) subjects to seek emergency medical care. Of these, 14 (53.8%) were diagnosed in the medical record with an opioid-associated, clinically relevant event. Three (11.5%) subjects were diagnosed with a pulmonary embolism with patients reporting only the low S_pO_2__ alarm as the driving force for seeking medical care. An additional 18 (5%) of subjects requested a follow-up sleep study from their primary care physician owing to low S_pO_2__ alarming. Importantly, in one case, the low S_pO_2__ alarm alerted the caregiver of a potentially critical OIRD event and by having been administered naloxone may have avoided a serious injury or even death (Table 3).

**Table 3.** Emergency department visits and opioid-related and non-opioid-related diagnoses with home monitoring

	Subjects (*N =* 359)
Sleep study	18 (5.0)
Emergency department visits and diagnosis	26 (7.2)

**Subjects With Emergency Department Visits and Emergency Department Documented *Opioid-related* Discharge Diagnoses *n =* 26**	
Opioid-induced cardiac ischemia	6 (23.1)
Opioid-induced neurologic ischemia (eg, stroke, seizure, transient ischemic attack)	4 (15.4)
Opioid-induced renal ischemia	1 (<1)
Opioid induced hypoxia/dyspnea/impending respiratory failure	3 (11.5)
Total	14 (53.8)

**Subjects With Emergency Department Visits and Emergency Department Documented *Non-Opioid-Related* Discharge Diagnoses *n =* 26**	
Non-opioid-related pulmonary embolism	3 (11.5)^a^
Other (eg, wound issues, pain control, bronchitis, vocal cord paralysis)	11 (34.5)
Total	12 (46.2)

^a^ Non-opioid-related event; however, all 3 subjects reported no symptoms but visited the emergency department because of frequent audible alarming of pulse oximeter. Data shown as *n* (%).

## Discussion

We utilized home capnography (P_ETCO_2__) and pulse oximetry (S_pO_2__) to monitor ventilation and oxygenation, respectively, in subjects undergoing orthopedic surgery. Prior studies have utilized the commercially available devices for continuous pulse oximetry monitoring in a cardiac surgical unit. The specific device used for this study uses technology for continuous pulse oximetry monitoring that has been used in general and surgical care units. We used both pulse oximetry and capnography in this study; however, there were no FDA-approved pulse oximeters for extraction of frequency for home use during the time of this study. Pulse oximetry and capnography offer 2 useful noninvasive techniques to facilitate respiratory monitoring in the hospital setting. The frequency data are extracted via the P_ETCO_2__ sampling line. Issues with condensation in the P_ETCO_2__ line resulted in less reliable actionable low and high P_ETCO_2__ and low frequency alarms compared with S_pO_2__ in the home setting. Continuous S_pO_2__ monitoring was the most reliable indicator of respiratory depression/compromise in the home setting. This study demonstrates that continuous S_pO_2__ monitoring with an escalating algorithm for response to alarms is a feasible intervention for preventing impending respiratory failure/compromise in the home.

Five key findings for quality improvement were identified. First, current processes for presurgical identification of positive airway pressure compliance in subjects previously diagnosed with obstructive sleep apnea were found to be suboptimal. Second, 25 (7%) of the total study population experienced ≥50 oxygen desaturation index per hour. Ten or greater oxygen desaturation index per hour have been correlated with sleep-disordered breathing owing to potential airway obstruction. Further study is needed to determine the relationship between the oxygen desaturation index and opioid morphine milligram equivalents. Third, subjects receiving interscalene nerve blocks with >90 morphine milligram equivalents per day required an increased need for supplemental oxygen, which was found to be statistically significant. Phrenic nerve paralysis as a complication may potentiate the effects of central acting opioids and requires further study. Fourth, despite deploying opioid sparing practices, the morphine milligram equivalents per day prescribed by health care providers are higher than recommended by the Centers for Disease Control and Prevention. Improved standardization and monitoring of prescribing practices should be considered. Fifth, 70% of subjects in this study were prescribed home oxygen prior to discharge who did not require it before surgery. This could potentially be avoided with improved prescreening measures and additional opioid-sparing regimens.

This study has several important limitations. Technology was employed in the uncontrolled home environment. Trained health care staff were not available to troubleshoot malfunctioning equipment in the home. This became apparent with the condensation in the P_ETCO_2__ sampling lines impeding the accurate measurement of P_ETCO_2__ and frequency. Despite using the pulse manufacturer’s cannula with moisture wicking capabilities, capnography was unreliable in the home setting owing to water condensation, which resulted in erroneous P_ETCO_2__ and breathing frequency. This is a significant issue considering the current recommendations of using capnography when monitoring patients on supplemental oxygen.

The initiative protocol was designed to identify only high-risk patients for opioid-related respiratory complications; thus, the utility of this monitoring strategy for all ambulatory surgical subjects has not been determined. The surveys administered to subjects and caregivers were not rigorously validated and should be viewed as hypothesis-generating. The study population was overwhelmingly white and may not be applicable to other ethnic groups. Finally, the COVID pandemic occurred during the initiative time frame and did impact study enrollment and the ability to approach all patients who met the inclusion criteria. Further impact of the pandemic was the fact that surgical procedures were ceased for 6–8 weeks the hospital became the overflow facility for full-capacity hospitals. Shared rotating staff resulted in a dilution of experienced research team members who had been trained in all aspects of the initiative to include enrollment referrals.

## Conclusions

Patients at risk for clinically relevant opioid events may experience higher rates of postoperative complications, hospital readmissions, clinically relevant events, or death. Strategies should be employed to identify patients preoperatively who are at high risk. Continuous monitoring of S_pO_2__ should be employed as a safety measure in high-risk subjects in the home postoperatively to facilitate earlier identification and intervention. Policy and reimbursement strategies should be created to support the use and reimbursement for short-term, continuous home monitoring of high-risk opioid subjects. This can be in the setting of postoperative care, acute pain, and/or chronic pain management. A second phase, larger scale study utilizing continuous S_pO_2__ and frequency obtained from S_pO_2__ plethysmography with centralized home monitoring utilizing wearable technology is planned.
