# Supplementary material for: Home Monitoring of High-Risk Individuals Receiving Opioids Post Orthopedic Surgery
Source: Respir Care. 2025 Aug 4;70(8):968–78. doi: 10.1089/respcare.11783 (PMC12411404; doi:10.1089/respcare.11783)
Supplement: Supplementary Data S1 [file respcare.11783_supplementary_datas1.docx]

**Supplementary Materials**

**The Orthopedic Specialty Hospital High-risk Opioid Subjects Home**

**Monitoring Protocol**

**
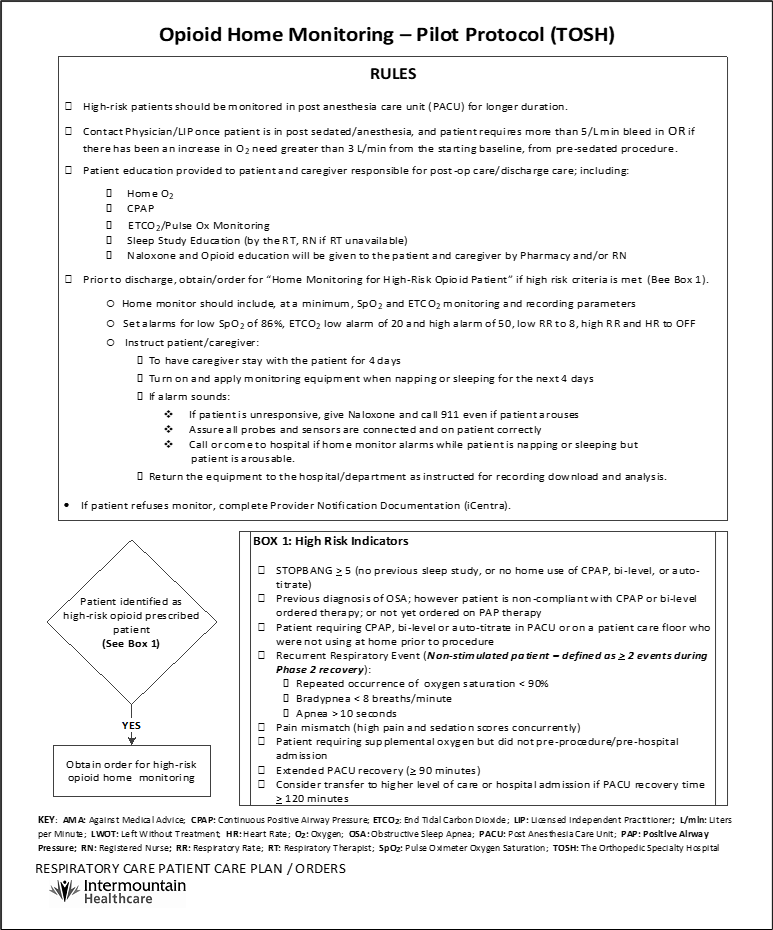
**
